# The Woes of a Stuffy Nose: A Case Report of Allergic Fungal Sinusitis

**DOI:** 10.5811/cpcem.2020.4.46866

**Published:** 2020-10-26

**Authors:** Tyler Lopachin, Grace Landers

**Affiliations:** Naval Medical Center Portsmouth, Department of Emergency Medicine, Portsmouth, Virginia

**Keywords:** Headache, sinus pain, congestion, stuffy nose, eye pain

## Abstract

**Introduction:**

Allergic fungal sinusitis (AFS) is a relatively uncommon cause of sinus pain and congestion. Extreme cases may require specialty evaluation and surgical treatment.

**Case Report:**

In this case, an otherwise healthy young man presented to the emergency department with sinus pain and congestion for two weeks and was admitted to surgery for resection of his AFS.

**Conclusion:**

This case demonstrates how a thorough history and physical exam can help catch potentially serious diseases, such as allergic fungal sinusitis, from the frequently benign chief complaint of sinus pain.

## INTRODUCTION

Sinusitis is one of the most common complaints in the country, developing in 12.5% of the entire United States population during a given year.^1^ The vast majority of these cases are caused by benign, self-limiting viral infections.^2^ Treatment in the emergency department (ED) revolves around symptomatic care with decongestants.^2^ Certain aspects of the history and physical exam, however, should raise the alarm for a more serious diagnosis. High-risk historical features, such as a diabetes, recent trauma, or immunocompromised status, or concerning physical exam findings such as pain with extraocular movement and proptosis warrant additional imaging or laboratory testing.

## CASE REPORT

The patient was a 27-year-old male without past medical history who presented with sinus congestion for two weeks. He stated that he had issues with sinus congestion many times before but this episode appeared to be more extreme than previous ones. He had been seen by his primary care doctor twice and given guaifenesin and pseudoephedrine for symptomatic relief. The night of presentation he complained of a headache that had gotten worse overnight with increasing sinus pressure. He denied fevers, chills, or blurry vision. The remainder of his review of systems was negative. On physical exam, vital signs were within normal limits and he was afebrile. Notably, there was mild right periorbital swelling along with multiple right nasal polyps on physical exam. In addition, he exhibited marked tenderness to palpation of right frontal, ethmoid, and maxillary sinuses. His extraocular movement was intact but notable for pain with vertical gaze in his right eye and diplopia. Otherwise, he demonstrated a normal physical exam with a non-focal neurological exam.

Initial laboratory evaluation that included complete blood count and basic metabolic panel was unremarkable. A computed tomography was ordered due to concern for the patient’s pain with extraocular movements and demonstrated destruction of the medial orbital wall and compression of the right superior rectus muscle (Image). The patient was diagnosed with allergic fungal sinusitis (AFS) with possible superimposed bacterial infection. He was admitted to the hospital under the otorhinolaryngology service with ophthalmologic consultation. On hospital day one a polypectomy was performed with debridement of the fungus. The patient did well postoperatively and was discharged home with doxycycline and outpatient follow-up.

## DISCUSSION

This case highlights an unusual etiology of an extremely common and often benign complaint—nasal congestion. However, as discussed earlier, there are a few parts of this patient’s history and physical that raised concern, specifically symptoms lasting two weeks without relief in spite of appropriate symptomatic care. The patient’s lack of improvement suggests a more serious etiology. Monocular pain with vertical gaze was perhaps the most troubling finding, especially in the presence of periorbital swelling. Often pain with eye movement is associated with orbital cellulitis, but it can occur with an intraorbital process that disrupts the function of the extraocular muscles. Finally, the patient’s nasal polyp disease and history of frequent nasal congestion are associated with his ultimate diagnosis, AFS.

Seen in patients with nasal polyps and asthma, AFS occurs when an airborne fungus begins to colonize the sinuses, leading to a predominately eosinophilic inflammatory response that causes thick mucus and debris to block the sinus spaces.^3^ The most commonly implicated species of fungi are Bipolaris, Curvularia, Aspergillus, Exserohilum, and Drechslera, all of which can infect an immunocompetent healthy host.^4^ In turn, eosinophilic degranulation products cause destruction of nasal mucosa and surrounding bony structures. Ultimately, this leads to bacterial colonization and penetration into the orbit itself, worsening clinical symptoms and inflammatory response. If untreated, as in this patient, localized destruction and intraorbital invasion occurs.^5^

While some patients can be medically managed with decongestants, the mainstay of treatment for patients who have failed outpatient management is endoscopic surgical removal of the fungus. It not only aids in the diagnosis, but allows the removal of the affecting material. This, in turn, provides better access for the administration of topical steroids postoperatively.^6^ Patients are also treated with systemic steroids both before and after the surgery. While preoperative dosing is more controversial, postoperative dosing consists of 0.5 milligrams per kilogram daily of prednisone and tapering down over the course of many weeks.^7^ Unfortunately, recurrence is common, and patients may need repeat surgical removal or long-term immune modulation; however, the latter is more controversial.^3^

CPC-EM Capsule

What do we already know about this clinical entity?

*Allergic fungal sinusitis is a disorder involving fungal colonization of the sinuses and subsequent inflammatory response in immunocompetent patients*.

What makes this presentation of disease reportable?

*This is a case of a serious diagnosis that required surgical management presenting in the emergency department as a common, often-benign complaint, sinusitis*.

What is the major learning point?

*Pain with extraocular muscle movement in the setting of sinus pain or headache should prompt further evaluation*.

How might this improve emergency medicine practice?

*This case highlights the importance of good physical exam and history taking, and provides some useful red flags when evaluating a patient with sinusitis*.

## CONCLUSION

While sinus pain and congestion are common presenting symptoms in the ED, this case demonstrates how even seemingly benign chief complaints can lead to potentially serious diagnoses. This case illustrates the importance of good history and physical exam skills for emergency physicians. It also highlights some key red flags to recognize in a patient with sinus pain.

## Figures and Tables

**Image:**
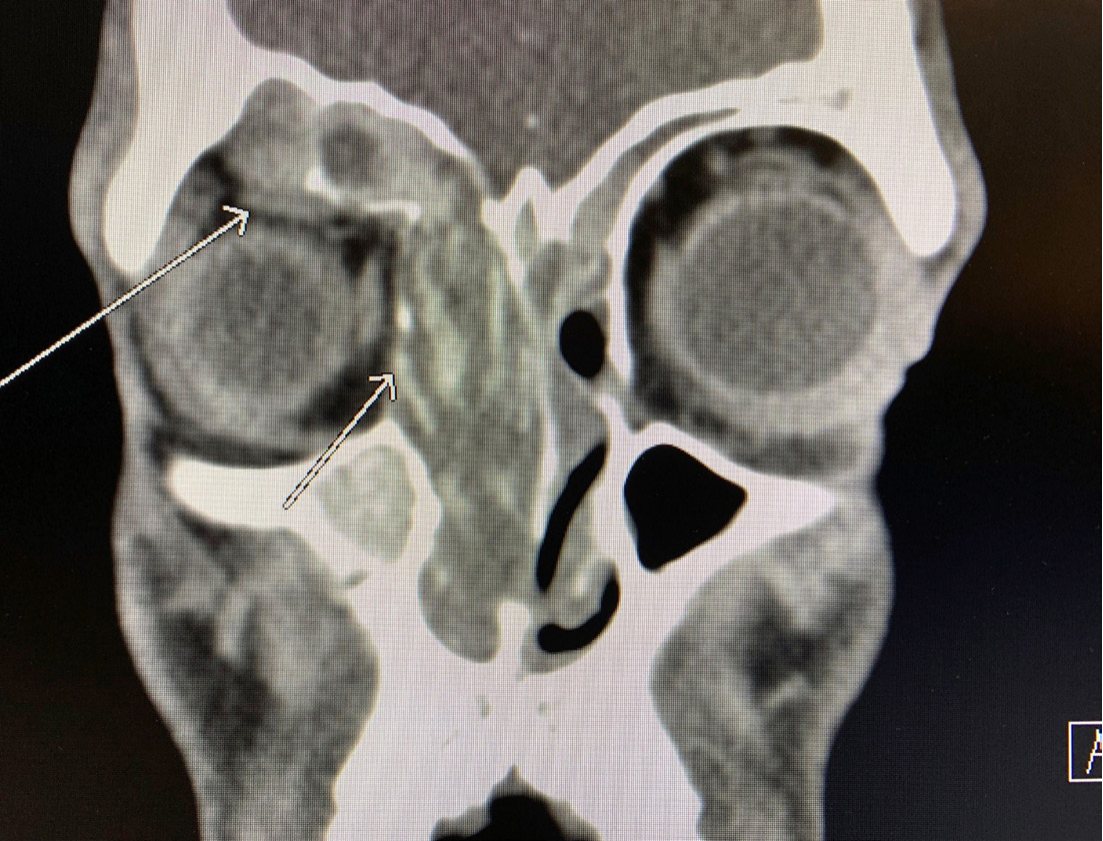
Computed tomography demonstrating allergic fungal sinusitis. The shorter arrow notes destruction of orbital wall, with longer arrow demonstrating compression of superior rectus muscle.
